# Applications of Recycled and Crushed Glass (RCG) as a Substitute for Natural Materials in Various Fields—A Review

**DOI:** 10.3390/ma16175957

**Published:** 2023-08-30

**Authors:** Cristian Epure, Corneliu Munteanu, Bogdan Istrate, Maria Harja, Florentin Buium

**Affiliations:** 1 Mechanical Engineering, Mechatronics and Robotics Department, “Gheorghe Asachi” Technical University of Iasi, 700050 Iasi, Romania; cristian.epure@student.tuiasi.ro (C.E.); bogdan.istrate@academic.tuiasi.ro (B.I.); 2 Technical Sciences Academy of Romania, 26 Dacia Blvd., 030167 Bucharest, Romania; 3 Chemical Engineering and Environmental Protection “Cristofor Simionescu”, “Gheorghe Asachi” Technical University of Iasi, 700050 Iasi, Romania; maria.harja@academic.tuiasi.ro

**Keywords:** glass, recycled glass, crushed glass, sand, aggregate, glasphalt, concrete, brick, filter

## Abstract

Glass is a substance that is present in most houses since glass-based items are made and consumed in relatively high quantities. This has led to the buildup of glass in concerning quantities all over the world, which is a problem for the environment. It is well known that glass has several advantageous physiochemical features that qualify it as an appropriate material for use in the construction industry as an aggregate. The features include being non-biodegradable, resistant to chemical assault, having low water absorption, having high hydraulic conductivity, having temperature-dependent ductility, having alterable particle gradation, and having a wide availability in a variety of forms and chemical compositions. Because of these qualities, glass has been used in various investigations and field tests conducted in civil engineering to evaluate its effectiveness as an engineering aggregate and to develop environmentally friendly management strategies for waste glass. These studies and research have utilized glass in various forms, such as fine recycled glass, medium recycled glass, coarse recycled glass, powdered glass, and glass-based geopolymers. This study focuses on research studies that present results on physicochemical, mechanical, and durability characteristics. These studies and research contain samples of pure glass or glass as replacement percentages in materials (0–100%), including but not limited to unbound granular materials (such as recycled concrete aggregates and crushed rock). In light of the information assembled in this review article, it is legitimate to claim that glass has strong promise as a material in various civil applications.

## 1. Introduction

### 1.1. Short History

The earliest glass ever discovered in its natural condition is what’s known as volcanic glass. This kind of glass was formed when lava was rapidly cooled. People fashioned spearheads and jewelry out of it and subsequently utilized the spearheads in hunting. Despite this, it is believed that the production of glass began in Egypt around 1400 BC. This exhibit details the whole process of creating and processing glass, down to the equipment and machines available. After the Egyptians taught the Phoenicians how to make glass, the Phoenicians continued the trade by sending decorations, vases, and flasks made of glass all across the globe. Perhaps it was also for this reason that people at first believed that they were the ones who found glass—another pivotal period in the development of glass occurred around the turn of the 20th century. A Roman artisan came up with the idea for a long, thin iron pipe with a tiny bulge at one end and a wooden extension at the other, and they used it to blow air through. Because of this, a technique known as blowing glass came into being and remained used for a significant time [1,2].

It is stated that Venice was once a significant center for the production of glass. The glass experts recruited to work here were sequestered on the island of Murano so they would not expose the process used to produce glass. Murano glassmakers were also responsible for the creation of the very first glass mirrors in the 12th century. Giorgio Agricola, a well-known artisan from Venice, is regarded as the “father” of the technical aspects of glassmaking. He detailed the procedure for producing glass in the year 1500 for us. The procedure of burning came into usage in the 16th century, after the technique of blowing, primarily throughout the 17th and 18th centuries, particularly for the production of glass, tiles, and pipes. The technique involves simply pushing the glass into the shape of a strip using a set of rollers, which pull the strip at a high speed while simultaneously cooling it gently for stress relief.

Even though lead crystal was an English innovation, the first plant in France to create lead crystal was the St. Louis factory in 1784. Later, in 1823, the famed Baccarat crystal factory came into being. Both factories are located in France. Sir Alastair Pilkington created float glass in the United Kingdom between 1952 and 1957. The production of float glass begins with the glass being heated to a temperature of around 1000 degrees Celsius, at which point it is molten and is poured continuously from the furnace into a shallow bath of molten tin. It disperses evenly throughout the can’s surface as it floats on top of it. Following the annealing process, the glass takes on the appearance of a polished product, with surfaces that are almost perfectly parallel. Shortly later, the same Sir Alastair Pilkington was a pioneer in the technique of pyrolytic deposition. This approach is used in production and involves spraying metal compounds onto glass while heated to a high temperature. This approach is perfect for insulating against the heat.

The aim to increase the quality of glass and its physical attributes led to the invention of tempered glass in the middle of the 20th century. This is accomplished by heating the material in a toughening furnace to around 700 degrees Celsius and then rapidly cooling it. This process brings the tensions inside the glass to a state of equilibrium, which endows the glass with unique properties such as very high resistance to intense mechanical impact and stability when subjected to changes in temperature. If it breaks, the object disintegrates into relatively few fragments, which significantly lowers the probability of suffering an injury. When it comes to interior doors, partitions, glass furniture, glass shower cubicles, elevators, stairs, store windows, and commercial premises, fully toughened glass is the material of choice [1,2].

### 1.2. Source of Prime Material

Waste is becoming an increasingly critical issue worldwide, both from an environmental and an economic point of view [3]. The practice of landfilling garbage has evolved into a serious environmental issue [4] due to the large quantities of waste products produced annually. The landfills are considered limited resources. In addition, the construction of roads and other transportation-related businesses are responsible for using significant quantities of natural resources. Incorporating recycled materials into construction projects like concrete, asphalt, ceramic tiles, thermal insulation, ecologically friendly pavements, etc., is one technique to reduce the negative effects on the environment. Commonly utilized paving materials include sand, gravel, crushed stone, asphalt, and concrete, in addition to other naturally occurring construction materials. In 2018, the global CO2 emissions connected to final energy consumption were responsible for 36% of the world’s total [5]. The building and construction industry was responsible for 36% of the greenhouse gas emissions. To maintain a healthy and sustainable environment, there has been a great need to investigate sustainable alternative materials that have comparable performance to typical building aggregates [6].

**Figure 1:**
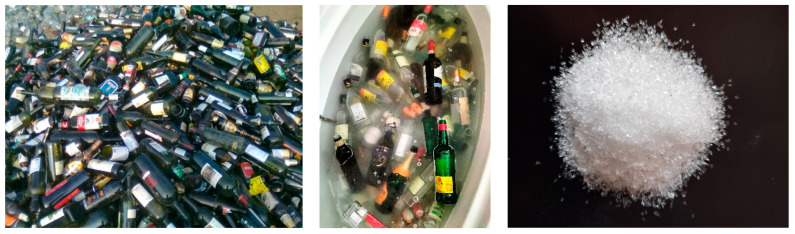
Recycled and crushed glass (RCG).

Glass is a component of many different types of products that most people use regularly and have gotten used to. It is possible for it to take the shape of a mirror, a glass bottle, the screen of a mobile device, the screen of a computer, the windows or doors of a house or automobile, or a table. Consequently, the world’s population continues to consume significant quantities of glass, some of which ultimately winds up as waste glass and contributes to generating about 130 million tons of waste glass each year [7]. In addition to that, reports indicate that recycling rates are rather poor. Only 13% of the glass produced in mainland China and 28% of the glass produced in the United States is recycled, respectively, resulting in about 40 and 11.54 million tons of waste glass produced each year. The years 2018–2019 saw Australia utilize over 1.21 million tons of glass annually, with approximately 90% of that usage going toward glass packaging. During the same period, it was estimated that the amount of waste glass created in Australia was 1.16 million tons, with a recycling rate of 60% [8]. Due to the huge quantities created, poor recycling rates, and the non-biodegradable nature of glass, a large quantity of waste glass has collected in landfills, which poses a hazard to natural flora and animals. The quantity of waste glass produced in various nations is shown in Table 1, along with the percentages of recycling and disposal achieved in Figure 1.

**Figure 2:**
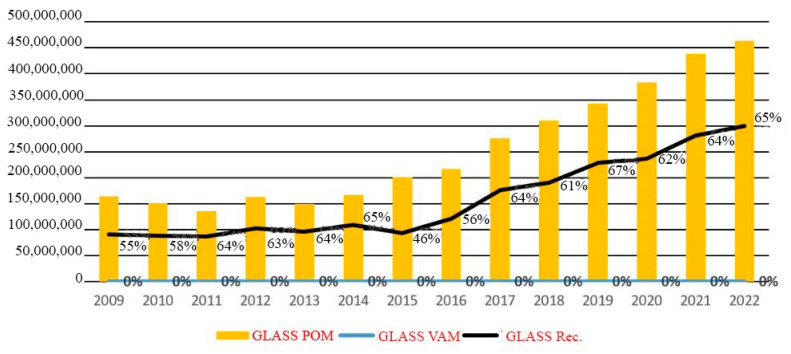
Evolution of glass recycling (kg) [11].

In Romania, in the evolution of packaging put on the market and recovered in the period 2009–2022, statistically, out of approximately 2.4 million tons of packaging put on the market in 2022 (POM—Put On Market), 1.5 million tons of packaging (61%) were recycled and 4% were recovered by other methods (VAM). This information comes from the Romanian Packaging Industry Association. During the year 2022, there were 463,400 tons of glass packaging placed on the market. Of that total, 65 percent was recycled. It is possible that by the year 2022, Romania will have achieved its previous (from 2008) glass recycling objectives of 60%, and the country is getting extremely to achieving its targets for the year 2025, which are 70% for glass, Figure 2 [11].

It has been determined that the civil engineering and construction industry is one of the fields that can greatly cut down on the troublesome buildup of glass waste at disposal sites. This has a good effect of lowering the quantity of glass headed to landfill sites and improving the natural environment by minimizing the usage of raw mined materials in engineering endeavors [10]. The industry includes projects that may use considerable volumes of glass. Glass aggregates may be found in a variety of forms, such as crushed glass, recycled glass, waste glass, fine recycled glass, medium recycled glass, coarse recycled glass, glass powder, foam glass, and glass geopolymers. These kinds of glass aggregates were used in the research studies and field testing. Glass cullet, also known as crushed recycled glass, is made up of waste glass that has been accumulated in recycling facilities and that has been mechanically crushed to a maximum particle size of 4.75, 9.50, or 19.00 mm, respectively. On the other hand, glass powder is made up of glass that has been mechanically crushed to a uniform consistency, with a specific size depending on the purpose for which it is being used. Mixing glass powder with an alkaline solution is typical for producing glass-based geopolymers. With this method, the rich silica environment in the glass powder helps stimulate the geopolymerization process.

It is also doubtful that substituting up to 10% of natural aggregates with waste glass will have substantial detrimental impacts on the overall performance of the mixes [12]. This is because natural aggregates are much denser than waste glass. RCG is an excellent option for usage as a supplemental material in various applications since it has a low impact on the surrounding environment [13]. It has been reported that cement-treated RCG mixes might benefit from adding up to fifteen percent of crushed recycled glass [14]. Because of its hydrophobic nature, waste glass seldom experiences significant changes in its maximum dry density in response to shifts in relative humidity. According to many studies, it is possible to integrate up to 30% of waste glass into products while meeting all standards [15].

It was discovered that recycled fine glass had appropriate strength for pavement foundation courses and excellent workability when added to waste rock aggregate at an optimal rate of 15% of dry mass [16]. When added to crushed recycled concrete at the same rate, the mix indicated promise for usage in pavement sub-base layers [17].

**Figure 3:**
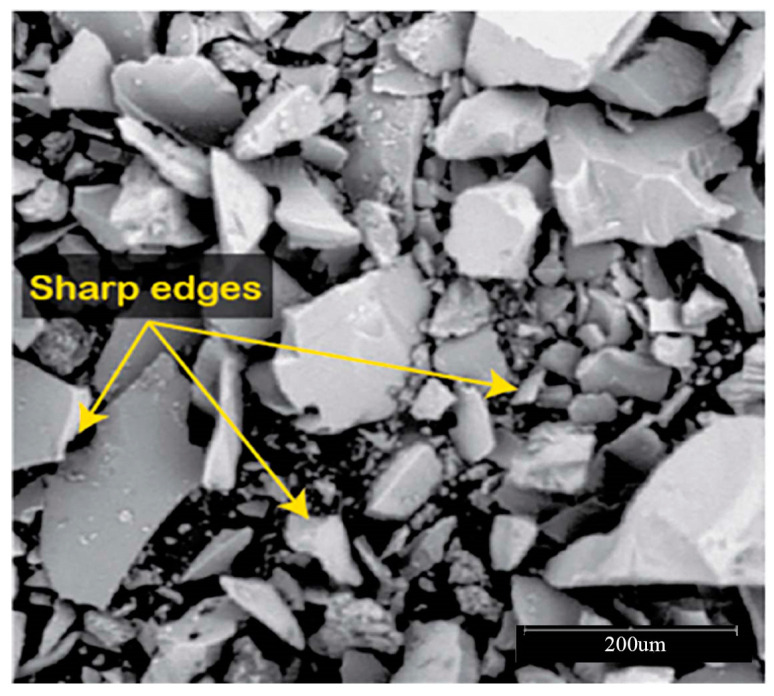
SEM micrographs of crushed waste glass powder (200 μm) [20].

The efficiency of a geopolymer based on recycled glass powder (RGP) in treating clay soils has been the subject of research [18]. It has been shown that the molar ratio of the alkali solution, beginning synthesis temperature, and polymerization time all play a role in the features of RGP geopolymer and its effectiveness in stabilizing clayey soils. RGP is an excellent ingredient for the alkali-silicon reaction (ASR) in RGP-based geopolymer cement [19] because it has a high concentration of silicon and is thus a rich supply of this element, Figure 3.

As researchers operating in the modern world, it is essential that all research carried out be consistent with the concept of sustainability. Our will makes it possible to protect valuable and dwindling natural resources for use by people living on our planet in the foreseeable future. A strategy of this kind not only helps to protect these resources but also has positive effects on the environment in the form of lower emissions of greenhouse gases, which in turn leads to a wide variety of positive effects on the environment.

## 2. Benefits Resulting from the Recycling of Glass Waste

The process of making glass materials and methods begins with the extraction of sand, which is often done in large quantities. In principle, glass may be recycled many times before the material’s quality suffers any damage. Before cullet can be utilized as a feedstock for the glass industry, it must go through several time-consuming and labor-intensive phases. These processes include separation, the removal of dirt, and cleaning. This presents a significant challenge for the reuse of cullet in glass production [21]. Table 2 shows the chemical composition of the most common recycled bottles [18].

Recycling glass would directly help the environment by saving natural resources and taking pressure off landfills. Additionally, it would have indirect positive effects in the form of savings in energy and money, for example, by prolonging the lifespan of industrial equipment such as furnaces. In addition, using waste glass may reduce the quantity of raw materials that need to be transported, resulting in less wear and strain on the transport routes [21]. According to the findings of their research, substituting glass for raw materials results in a reduction in the needed furnace temperature, which results in energy savings. In particular, the hazardous impacts of manufacturing and disposing of glass may be reduced by at least 30% if the glass is recycled rather than disposed of in a landfill. Glass may be recycled, which helps keep important materials out of landfills and saves money. Similarly, the Glass Packaging Institute conducted a life cycle assessment (LCA) of container glass in North America. They discovered that recycling glass at a rate of 50% would eliminate two million and two hundred thousand metric tons of carbon dioxide from the environment. This is the same as taking approximately 400,000 automobiles off the road for an entire year and eradicating their associated CO_2_ emissions [22].

## 3. Materials and Methods

### 3.1. Properties of Glass

#### 3.1.1. Chemical Properties

Glass is available in various shapes, colors, and hues, each with unique chemical makeup. One of the key reasons why glass is not remanufactured is because different colored glasses have different chemical characteristics. This prevents the glass from being reused. The chemistry behind some of the most prevalent forms of commercial glass is laid out in Table 3 [23,24,25].

In its most basic form, glass is an amorphous substance composed of a combination of metal oxides that have been quickly melted and cooled to create a solid structure. Glass is typically resistant to chemical attack and does not react with the majority of commonly used chemicals. Because it does not react with chemicals, glass is ideal for use in applications such as packaging, laboratory utensils, and other places where chemical resistance is required.

#### 3.1.2. Physical Properties

Table 4 lists various physical characteristics of crushed waste glass and various physical characteristics. The calculation for bulk density, also known as the loose bulk density, involves dividing the weight by the volume of the material in question. The mass of particles that have a ratio of length to thickness that is greater than three is referred to as the shape index, and it is given as a percentage of the total dry mass of the particles that were tested. The fineness modulus may be calculated by adding the total percentages of a sample of aggregates retained on each sieve in a certain series and then dividing that number by 100 [26]. The flakiness index is the proportion of particles, by mass, whose least dimension is less than three-fifths of the mean dimension. This percentage is expressed as a fraction [27,28].

### 3.2. Recycled and Crushed Glass Usage as Fine Aggregate in Concrete

It was projected in 2010 that more than 12 billion metric tons of concrete would be manufactured worldwide [29], which has been gradually climbing since that year. As a more environmentally friendly alternative to conventional aggregates like sand and gravel, the use of glass as a substitute for these materials is gaining popularity as a construction product choice. In relation to the usage of crushed glass as a fine aggregate in concrete, the following are some significant issues to consider.

Material characteristics: because of its material features, crushed glass may be used as an acceptable substitute for fine aggregates, its particle size distribution is comparable to that of natural sand, and it has the capability of imparting a smooth surface texture to concrete. 

Environmental Benefits: the use of broken glass in concrete helps to lower the demand for natural resources like sand and gravel, it can help reduce litter and promote sustainable practices by using recycled glass.

The capacity to be worked and mixed: To maintain the correct level of workability in concrete mixes that include broken glass, it may be necessary to make some slight modifications to the ratio of water to cement, to obtain the intended level of strength and durability, it is essential to check that the concrete mix has the appropriate proportions of each ingredient. 

Glass is considered to be a pozzolanic material, which implies that it is capable of reacting with calcium hydroxide in the presence of moisture to generate additional cementitious compounds. This property is what gives the glass its pozzolanic classification. This reaction, also known as pozzolanic activity, may contribute to concrete’s durability and tensile strength. Considerations Regarding Durability: Adding broken glass to concrete may impact the material’s capacity to withstand wear and tear over time. Compared to cement paste, glass has a greater coefficient of thermal expansion, which increases the risk of breaking and lowers the material’s durability. It is necessary to implement a suitable mix of design and quality control procedures to cut these hazards. Various research studies have evaluated the performance of concrete containing crushed glass.

#### 3.2.1. The Workability of Concrete after Having Fine Particles Substituted with RCG

In the study that de Castro and De Brito conducted on concrete that was made by substituting construction aggregates with a percentage rate of 0%, 5%, 10%, and 20%, they discovered that the size of the aggregate has a significant impact on the ability of concrete made with glass aggregates to be worked. This was the finding they came to after researching concrete made by substituting construction aggregates with a percentage rate of 0%, 5%, 10%, and 20%. To improve the workability of the concrete, the ratio of water to cement was raised to somewhere between 0.56 and 0.57, and twenty percent of the concrete was made up of fine recycled glass. There was a little decrease in the material’s fresh density whenever there was a larger amount of glass aggregate in the concrete mix. The compressive strength of the concrete began to decline at the same time as the percentage of glass in the concrete continued to rise. The water absorption of the concrete dropped by between 15 and 23% when fine glass aggregate was employed at replacement percentages of either 5 or 10%. The majority of the time, the incorporation of recycled glass into concrete does not result in a discernible weakening of the material as a whole [30].

According to Adaway and Wang’s [30] findings, the slump of concrete containing 15% and 25% RCG increased, whereas the slump containing 20%, 30%, and 40% RCG decreased. This was seen in both types of concrete. Ali and Al-Tersawy [31] used a procedure known as the slump flow test to assess the workability of self-compacting concrete. This test is performed by allowing the concrete to flow over a predetermined height. They realized that concrete containing RCG obtained slump flow values equivalent to those of the control while having lower dosages of the superplasticizer. In addition, the concrete made with 50% RCG as a substitute for fine aggregate achieved the maximum slump flow value of 740 mm, which was 12% more than the value obtained by the control. In addition, Borhan [32] tested the performance of concrete made with fine particles replaced with RCG and either 0.3% or 0.5% basalt fibers, depending on the quantity of the complete mix. According to the findings, an increase in the percentage of RCG replacement led to an improvement in the concrete’s workability. This was the case regardless of the amount of RCG substitution used. In particular, a concrete mixture of 60% RCG reached the maximum workability value for concrete that consisted of RCG. According to Taha and Nounu [33], the smooth surface and minute water absorption of RCG particles led to a reduced cohesive force in the concrete mixes, which resulted in segregation and bleeding of the concrete. This was found as a consequence of the smooth surface of the RCG particles.

Workability would suffer only a little from mild substitutions of up to 30%, according to Rajeev Devaraj and the other people who worked on this project. This would translate to a loss of around 22% in workability. It has been widely documented that the presence of glass components in concrete mixes has a detrimental effect on the mix’s capacity to be worked with. This is the case even when the glass components are quite small. Even if the sizes of the glass particles are smaller than the permissible size limit that is required by standards, the faceted nature of the glass particles and the irregular shapes will give increased resistance to the dynamic response of wet concrete mix to load, which would compromise the flowability of the concrete mix. This is because the irregular shapes would increase resistance to the dynamic response of wet concrete mix to load [34].

#### 3.2.2. Concrete’s Mechanical Strength 

Malik Lee et al. [35] and Adaway and Wang [30] found that concrete containing up to 30% RCG demonstrated compressive strength values greater than the control at 7 and 28 days. On the other hand, concrete with replacement levels that were more than 30% higher had a drop in compressive strength. In addition, after 28 days, concrete with replacement ratios of up to 30 percent exhibited compressive strengths that were higher than those shown by the control. In addition, Limbachiya [36] found that the compressive strength of specimens that included RCG decreased with increasing levels of RCG replacement when those levels were greater than 20%. This was the case when the replacement levels were above 20%.

In addition, Taha and Nounu [33] found that between 0 and 200 days after curing, the control specimens showed greater compressive strengths when compared to the specimens containing 50% and 100% RCG. This was the case regardless of whether the specimens were tested before or after curing. This was true regardless of how long the material was allowed to mature; the time it took to mature made no difference. After 6 days, the concrete made of 50.1% RCG in terms of compressive strength obtained a lower value than the concrete made of 100% RCG, which has a higher strength than that of the control. According to Castro and de Brito, the workability of concrete that contains RCG is strongly influenced by the particle size of the RCG. Based on the findings of experiments relating to mechanical strength, density, water absorption, and resistance to carbonation, concrete that contains partial RCG content is generally workable [25].

Chen et al. [37] observed the significant compressive strength of concrete in which sand was replaced with E-glass. In addition, Lee et al. [38] evaluated the compressive strength of concrete blocks that included RCG aggregate in various sizes, including unshifted, 2.36 mm, 1.18 mm, and 0.60 mm. They found that these blocks had a higher compressive str following studies conducted.

They discovered that after 28 days of curing, the only concrete blocks that attained compressive strength values superior to the control were those with less than 0.60 mm of RCG. In addition, Taha and Nounu [33] discovered that between about 0 and 200 days after curing, the control specimens had better compressive strengths when compared to the specimens containing 50% and 100% RCG. This was the case regardless of the curing time. According to Oliveira et al. [39], the mortar that included 20% RCG at a volumetric ratio of 1:5 exhibited the best performance, demonstrating appropriate levels of mechanical strength (both compressive and bending), water absorption, and cracking behavior.

**Figure 4:**
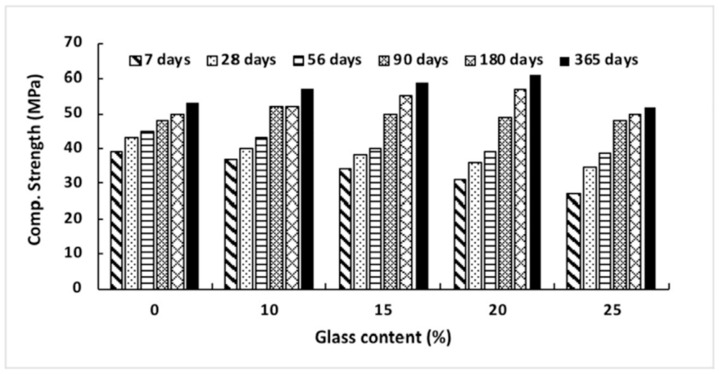
Compressive strength development in concrete with different glass powder content [40].

According to Ismail and Al-Hashmi’s [40] research from 2009, a large improvement in late-age strength may be accomplished by producing a denser and less permeable microstructure. This occurs due to the filling impact that sub-micron-sized glass particles have on the material. As seen in Figure 4, the compressive strength of the concrete declines with increasing volumes of glass sand up to 90 days after it has been mixed in. However, in the same mixtures, a small improvement in strength was seen with a glass sand substitution of up to 20% of the total volume. Mixes containing more than 20% glass sand showed no discernible weakening in strength compared to the control group. This might be because using waste glass powder to replace up to twenty percent of the cement or sand in concrete can increase the pozzolanic reaction and function as a filler material, filling the majority of the gaps between the big agglomerates in concrete. As can be seen in Figure 4, the kind of glass powder used in the production of concrete affects the material’s compressive strength. When recycled green glass powder was utilized in concrete as a partial substitute for cement in amounts up to 15%, the strength of the concrete was found to be significantly reduced. However, except for a 15% replacement rate, there was no significant difference in the strength of brown and neon glass powder. The high compressive strength seen at 13% of neon glass may be due to the large quantity of calcium carbonate (CaCO_3_), which has a significant impact on the compressive strength. This is because calcium carbonate is a substantial component of glass.

**Figure 5:**
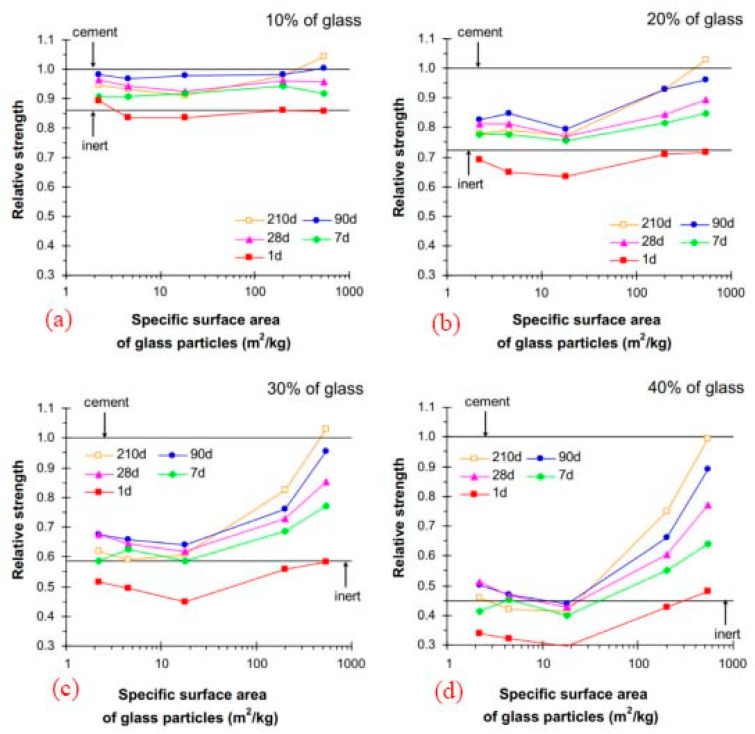
Strength comparison of mortars in terms of RCG fineness and curing time (**a**–**d**) [41].

According to the research of Mejdi et al. (2020) [41] and Lu et al. (2017) [41], the greater specific surface of recycled and crushed glass may contribute to the larger rise in compressive strength, and a longer curing time is also advantageous to the growth of strength. The degree to which recycled and broken glass dissolves and reacts depends on several elements, including size and curing time. The recycled and broken glass acted more like an inert filler on the first day of curing, but their pozzolanic capabilities began to manifest after seven days, Figure 5.

A higher ratio of water content in RCG aggregate mixtures, but also a weakening of the bond between glass aggregates and cement, led to a reduction in the compressive strength of concrete [42]. The researchers observed that the cement paste and glass aggregates lost their ability to bond with each other over time. 

#### 3.2.3. Flexural Strength

The studies indicated that the addition of fine RCG led to an increase in flexural strength in the same way that it led to an increase in compressive strength. On the other hand, the majority of investigations concluded that the incorporation of RCG resulted in a reduction in the material’s compressive strength. The flexural strengths of the concrete specimens that were assessed by Abdallah and Fan [29], Turgut and Yahlizade [43], Ismail and Al-Hashmi [40], and Batayneh et al. [44] were found to be greater than those of the control. This was the conclusion reached by all four research groups. In contrast, the concrete specimens that were tested by Park et al. [45], Taha and Nounu [33], and Ali and Al-Tersawy [31] had lower bending strengths than the control products. 

**Figure 6:**
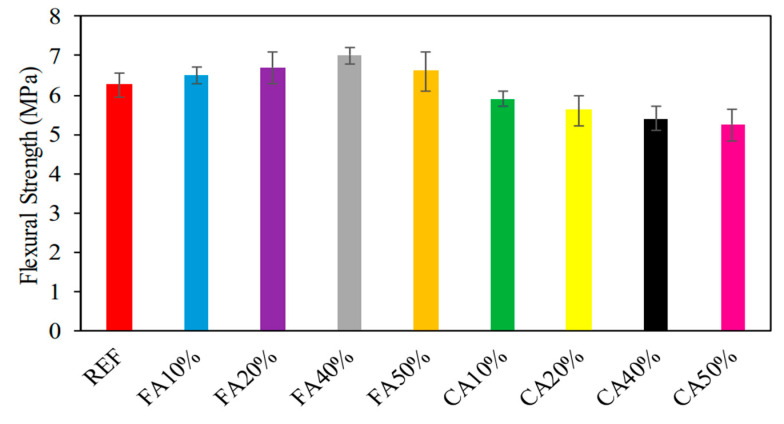
Consequences of FS [46].

The investigation that Ali ‘Ihsan Celik [46] and his colleagues carried out revealed that the types of absorbed strength varied, ranging from 5.2 MPa to 7.0 MPa. The results of the test to determine the flexural strength of the samples are shown in Figure 6. The flexural strength of the material is shown with varied percentages of CWG content in Figure 3, which may be found here. It was discovered that the flexural strength rose by 3.2%, 6.3%, and 11.1%, respectively, with the addition of CWG at 10%, 20%, 40%, and 50% of the changed fine aggregate. These percentages correspond to a 10%, 20%, 40%, and 50% composition of altered fine aggregate. The increase in toughness was quantified as a percentage that came out to 4.8%. In contrast, the flexural strength rose by 3.2%, 6.3%, 11.1%, and 4.8%, respectively, when measured in percentage terms compared to the sample that served as the reference (6.3 MPa). This may be attributable to the pozzolanic processes, which, as time passes, speed up, work against the hardening process, and lead to an increase in flexural strength. Additionally, Shehata et al. [47] observed a behavior associated with this one. 

According to Saribiyik et al. [48], using 30% glass aggregate powder as a substitute yielded the best results in terms of compressive strength as well as bending strength. When the amount of glass powder substitution was greater than 30%, there was a decrease in both the compressive and flexural strengths. The researchers, Saribiyik et al. [48], suggested determining the ideal replacement content for waste glass. Following the experiments carried out by Topcu et al. [49], it appears that the values of the bending strength decreased with the increase of WG. Another investigation by Arivalagan found [50] that the flexural strength of the specimen could be increased by 20% when glass powder was used as a substitute for fine aggregate in the mixture.

### 3.3. Properties of AAMs (Geopolymer) Containing RCG

Alkali-activated materials (AAMs) have been researched as the next generation of sustainable binding materials with much less embodied energy and a smaller carbon footprint than OPC [51]. AAMs are environmentally friendly due to the utilization of aluminosilicate wastes as precursors [52,53,54,55]. Because of the variety of raw materials, AAMs are as flexible and locally adaptive as the present toolkits of sustainable cements [54]. Activating the aluminosilicate precursors during the synthesis of AAMs requires using an alkaline solution such as NaOH, KOH, water glass, etc., and the reaction itself involves the dissolution of aluminosilicates and the formation of hydration products. The predominant binding phase of Ca-rich AAMs is known as the calcium (sodium) aluminate silicate hydrate (C-(N-) A-S-H), whereas in Ca- deficient reaction systems, the sodium aluminate silicate hydrate (N-A-S-H) would precipitate as the principal product [56].

It should be noted that the low-calcium AAMs are also called “geopolymers”. Due to the presence of magnesium, sulfur, iron, etc., in raw materials, numerous secondary phases may precipitate, such as M-S-H, hydrotalcite, AFt, AFm, Fe-hydrogarnet etc. However, the influence of each secondary phase on the material characteristics of AAMs should be the subject of more research. Generally, the high-calcium AAMs can harden at ambient temperature owing to the additional nucleation sites provided by CaO. On the other hand, the low-calcium AAMs often need a higher-temperature curing process. Compared to the SiO_2_ content of other aluminosilicate raw materials, the CaO and Al_2_O_3_ concentrations of RCG are relatively low, while the SiO_2_ content is quite high. Therefore, RCG is frequently combined with other Ca or Al-rich precursor materials to form AAMs (geopolymers) [55,56,57,58,59,60,61,62]. Many factors, including glass fineness, curing time, curing temperature, concentration of alkaline solution, and the properties of other raw materials blended with RCG, can affect the mechanical properties of RCG-based AAMs (geopolymers) [55,56,57,58,59,60,61,62]. In most cases, systematic strength testing is the best technique to discover the optimal method for manufacturing AAMs with appropriate mechanical characteristics.

The concentration of alkaline solution will always have a considerable impact on the progression of the compressive strength of any and all AAMs. If the reaction system does not have enough alkali, the aluminosilicate precursors cannot completely dissolve. This will have a significant impact on the formation of hydration products and will result in a reduction in the strength of AAMs [52]. On the other hand, excessive alkali will prevent the raw materials from dissolving owing to the electrostatic shielding that it would cause in the aqueous phases. As a result, the concentration of alkali that is optimal for all AAMs always exists. According to the findings of Xiao and collaborators [52], a solution of 5 M NaOH produced geopolymer with the maximum compressive strength (average size = 15.4 m).

**Figure 7:**
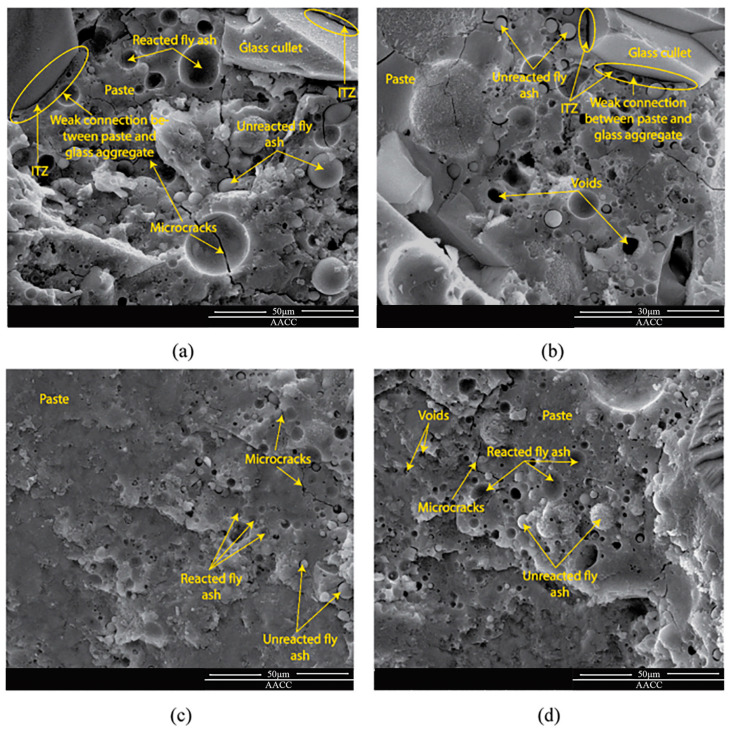
SEM of geopolymer mortar at 90 days (**a**) Glass (Ambient); (**b**) Glass (Oven); (**c**) Sand (Ambient); (**d**) Sand (Oven) [20].

These experiments by Hong Lich Dinh et al. [20] are noteworthy for several reasons. One is that the proposed equations for the mechanical features match the findings of earlier research. When the aggregate/binder ratio is greater than 2.5, they discover that geopolymer (GP) is unsuitable for buildings because it is too rigid. By using RCG in place of sand, the compressive and tensile strengths of GP were lowered by about 20–30% and 10–20%, respectively. Compressive strengths of 20–30 MPa and 35–46 MPa were achieved in RCG and OPC products after 28 days of curing at ambient temperature and higher temperatures, respectively. RCG products remained competitive with OPC in this regard. To make use of the full potential of waste glass, it is important to pay attention to the examination of the levels of impurity and reactive amorphous content within the waste materials. When using the ambient curing process, combining the two groups of sand and RCG led to a considerable increase in compressive strength (48–53%) from 7 to 28 days. It is possible to conclude that RCG has either no influence or very little effect on the growth of the geopolymerization process. There is a significant possibility that the use of geopolymers may mitigate the impact of cement manufacturing on climate change. A mix design and correlation equations for geopolymer concrete that do not depend on natural resources are analyzed by the researchers based on experimental data that was provided for 90 days Figure 7. Although the mixture does not include any cement or sand, the specimens have a strength equivalent to OPC concrete. These materials provide a highly feasible strength choice for precast constructions like bridges and buildings when oven-cured in RCG units. Examples of precast structures include bridges and buildings. In contrast, specimens treated by the ambient curing procedure are most appropriate for non-structural applications like patio slabs, sidewalks, and roadways.

Firas Abed Turkey et al. [62] evaluated the effect of temperature exposure on the characteristics of FA+GP geopolymer mortar and LWAGC. The research found that when geopolymers were heated to higher-than-normal temperatures, there was a discernible change in both their performance and their properties. The unexposed geopolymer mortar exhibited outstanding mechanical compressive strength due to the quick geopolymerization reaction rates at the high activated concentration. However, unexposed LWAGC had a comparatively low compressive strength because of the low strength and density of LWA in the geopolymer framework. Due to the fact that the curing process is still ongoing, the compressive strength of the geopolymer mortar heated to 200 °C increased by 8.52 percent.

**Figure 8:**
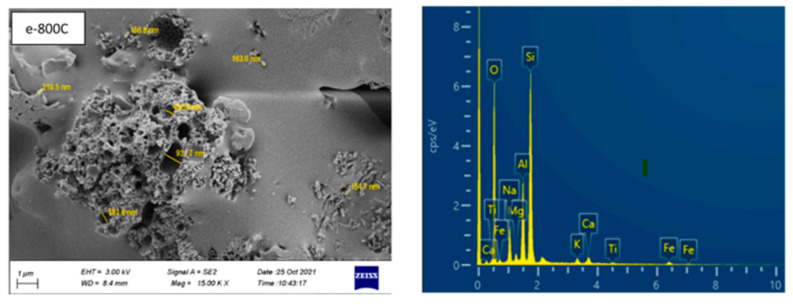
SEM Photo and (EDS) Spectrum Curves for The LWAGC [62].

On the other hand, its strength begins to diminish and degrade at a temperature of 400 °C, and it reaches its point of greatest degradation at 800 °C Figure 8, resulting in a loss of 37.3% of its original strength. Firas Abed Turkey et al. [62], who were involved, used a combination of glass powder, fly ash, and two different kinds of lightweight aggregates in our work. To enhance the performance of geopolymer concrete in future research, it may be possible to combine glass powder with several other kinds of alumina-silica sources. Additionally, it may be feasible to combine this powder with lightweight aggregates derived from natural sources, such as volcanic pumice, diatomite, and slag. 

**Figure 9:**
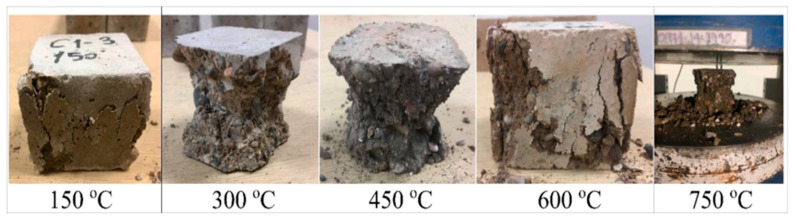
Fractured surfaces of samples exposed to high temperatures after compressive strength test [62].

Aslhan Nida Derinpinar et al. [63] conducted research in 2022 to investigate the behavior of geopolymer concrete at increased temperatures based on the replacement of slag with glass powder. At 28 days, the findings indicated that the sample with code G0 had the maximum compressive strength (39.54 MPa), whereas the sample with code G20 had the lowest compressive strength (30.67 MPa). The relative concentration gradient (RCG) of substitution in the mixes rose, which resulted in a drop in the sample strengths. The G0, G5, G10, and G15 coded mixture samples had splitting tensile strengths of 4.22, 3.81, 3.67, and 3.49 MPa, respectively. The G20-coded mixture sample had a value of 3.34 MPa. There is an exponential connection between compressive strength and splitting tensile strength, and the correlation coefficient values for this relationship were 0.91. The compressive strength of the sample tubes that were air-cooled throughout the thermal testing process showed an increase up to 300 °C, Figure 9. At temperatures of 450 °C and above, the reduction started to take place. In the case of samples that were cooled with water, a decrease in strength was seen at temperatures of 150 °C and above. The samples were impacted more badly by being cooled in water instead of in air. There was a direct correlation between the temperatures to which the samples were subjected and the amount of weight loss in the samples. The weight loss of samples that were heated to 750 °C varied from 9.82% to 10.30% for air-cooled samples and 11.59% to 14.29% for water-cooled samples.

The surface deformations of the samples that were cooled by water were much greater than those of the samples that were cooled by air. At the same time, when the temperatures rose, the hues of the samples became more muted. The microstructures of the samples were more degraded as the temperature rose. Cracks began to form in the structure of the geopolymer gel, especially when it was heated to temperatures of 450 or higher. Up to 300 °C, there was a decline in the ratio of silicon to aluminum in the samples, while there was a rise in the calcium to silicon ratio. At temperatures higher than 450 °C, the ratio of silicon to calcium decreased while the ratio of silicon to aluminum increased. Aslhan Nida Derinpinar et al. [63] revealed that, in general, the strength losses in samples subjected to extreme temperatures were reduced in inverse proportion to the rise in the RCG substitution ratio. The resilience of the concrete geopolymer to high temperatures was significantly enhanced due to RCG substitution.

**Figure 10:**
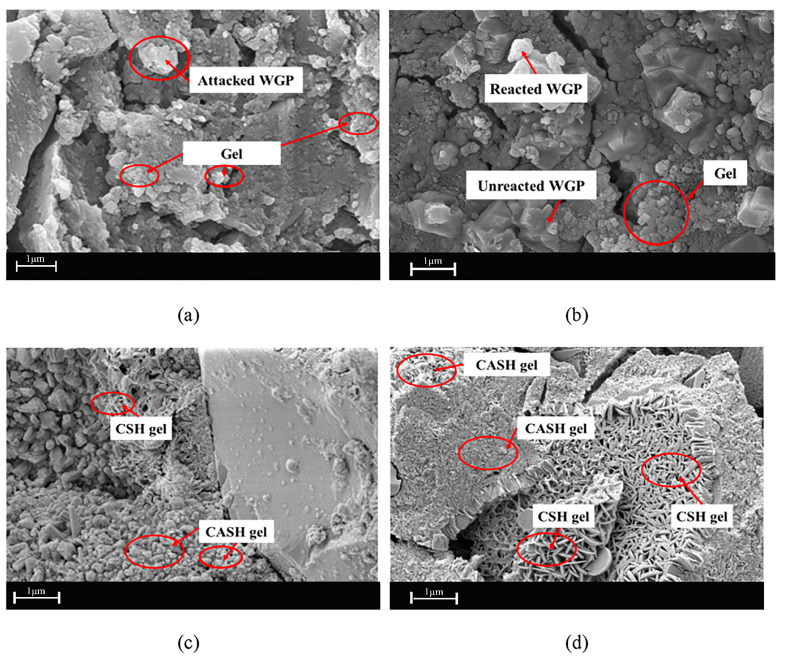
Microstructures of 100 mm cube sample on 28th day (80 °C, 50% RH): point 1 (**a**,**c**); point 2 (**b**,**d**) [65].

Xi Jiang et al. [64] investigated the impact size effects have on the qualities of a geopolymer paste built on slag and waste glass. According to the findings of the experiments, the negative influence on size that GBFS-WGP-based geopolymers have is considerable. The compressive strengths of geopolymer samples with sizes of 70.7 mm and 100 mm (the bigger size) fell by 12.9% and 36.2%, respectively, under conventional curing conditions of 20 degrees Celsius and 95% relative humidity. This was compared to the compressive strengths of the 50 mm (smaller size) cubic sample. Throughout the geopolymerization process, distinct phases call for using different aspects of relative humidity (RH%). At the beginning of the reaction process, water will dissolve the alkaline activator, which will help the reaction proceed. An uneven distribution of moisture inside the geopolymer led to an uneven distribution of moisture stress, resulting in a difference in compressive strength during the curing stage. Excessive moisture impeded the condensation process at this stage, which led to a difference in compressive strength. Wet curing plays an important role in producing dense matrix density, an alkali-induced reaction, and the endurance of the geopolymer during the curing stage. However, the compressive strength of GBFS-WGP-based geopolymers may decrease due to the loss of the alkali activator during the curing process when water saturation occurs. Because of this, it is essential to consider both the temperature of the polymerization process and the relative humidity (RH), both of which are significant for the geopolymer’s mechanical characteristics. The research by Xi Jiang et al. [64] shows that when the relative humidity was 50% Figure 10, the alkali activator did not react sufficiently. Consequently, the excess alkali activator crystallized on the sample surface over time, leading to a fall in PH. This was shown by the findings of the study. Because of the reduced PH, the carbonation resistance would decrease. Xi Jiang believes that to explore the influence of size, it will be essential for future research to include the construction of further AAM or geopolymer-based buildings at full scale. This will contribute to the practical use of a durable concrete process. Water will dissolve the alkaline activator, which will help the reaction proceed. An uneven distribution of moisture inside the geopolymer led to an uneven distribution of moisture stress, resulting in a difference in compressive strength during the curing stage. Excessive moisture impeded the condensation process at this stage, which led to a difference in compressive strength. Wet curing plays an important role in the production of dense matrix density, an alkali-induced reaction, and the endurance of the geopolymer during the curing stage. However, the compressive strength of GBFS-WGP-based geopolymers may decrease due to the loss of the alkali activator during the curing process when water saturation occurs. Because of this, it is essential to consider both the temperature of the polymerization process and the relative humidity (RH), both of which are significant for the geopolymer’s mechanical characteristics. The research by Xi Jiang et al. [64] shows that when the relative humidity was 50% Figure 10, the alkali activator did not react sufficiently. As a consequence, the excess alkali activator crystallized on the sample surface over time, which led to a fall in PH. This was shown by the findings of the study. Because of the reduced PH, the carbonation resistance would decrease. Xi Jiang believes that to explore the influence of size, it will be essential for future research to include the construction of further AAM or geopolymer-based buildings at full scale. This will contribute to the practical use of durable concrete. Ali Ihsan Celik et al. [65] used changes in the molarity ratios and percentages of RCG present in GPC. This was done to investigate the impact that RCG had on GPC when it was combined with fly ash in certain proportions. As a result, the molarity values of the NaOH dispersion investigated in the research were M11, M13, and M16. It was discovered that adding RCG decreased the workability of M11, M13, and M16 NaOH by an average of 17%, 10%, and 67%, respectively. This was determined by analyzing the data. According to the findings, the tailing values went down as the molarity went up. In addition, the impact of RCG % on tainting was investigated while the molarity was maintained at the same level throughout the study. It was discovered that the taint values dropped as the proportion of RCG rose in the mixture. However, when the proportion of NaOH was examined at 0%, 10%, 20%, 20%, 30%, and 40%, the ratio of fly ash to RCG in the concrete mix rose. Even though the proportion of fly ash to WGP in the concrete mix rose, it was discovered that there was a decline in the quality of the finished product. Ali Ihsan Celik et al. [65] say that using 10% WGP and 13 molarity NaOH together will give the best results regarding how long it takes to cure, how easy it is to work with, and how strong it is.

### 3.4. Glasphalt

In 1960, researchers substituted a percentage of the aggregates in an asphalt mixture with waste glass [65]. This began the concept later known as glasphalt, a means to reuse waste glass. The primary difference between hot mix asphalt (HMA) and glasphalt is that between 5 and 40 percent of the coarse aggregate or fine aggregate in glasphalt is substituted with broken glass. Glass is an inorganic and non-metallic substance that cannot be burned or broken down; hence, the reuse of waste glass for such applications is of utmost significance [66,67].

According to the findings of Airey et al. [68], an asphalt mixture containing 10–15% glass cullet can display reasonable performance. In that study, 2% lime was utilized as an anti-stripping agent, which is worth pointing out. According to the findings of Airey et al. [68], the maximum size of glass particles that are permitted for reasons of safety should not be more than 4.75 mm. After one year of usage, Su and Chen [69] found that asphalt specimens that included 10% broken glass had superior performance to ordinary hot mix asphalt (HMA). This was found in their research on asphalt specimens. Researchers led by Arabani [61] used the indirect tensile test technique to investigate the effects of temperature on the stiffness modulus of hot mix asphalt (HMA). According to their research findings, the fraction of RCG that should be used in asphalt to achieve the greatest possible rigidity modulus is 15%. Additionally, the correct RCG content often improves both the skid resistance of asphalt and the light reflection intensities, which contributes to an increase in overall driving safety. 

Interlocking and angularity of glass particles allowed glasphalt to have a greater stiffness modulus than conventional asphalt, as shown by Shafabakhsh and Sajed [66]. However, the stiffness modulus is reduced when using a glass content above 15% due to the increased sliding of glass particles on one another. The researchers found that specimens with a glass content of 15% had the maximum stiffness modulus when tested at a temperature that was held constant. At two constant loads of 250 and 400 kPa, fatigue life tests were carried out with hydrated lime acting as the antistripping agent. This modeling of the long-term fatigue performance of glasphalt under dynamic loading situations was done by Arabani et al. [67]. Four different percentages of glass were tested at five degrees Celsius. The performance of glasphalt under dynamic loading situations was done by Arabani et al. [67]. Four different percentages of glass were tested at five degrees Celsius, twenty-five degrees Celsius, and forty degrees Celsius: five percent, ten percent, fifteen percent, and twenty percent. Based on what was found, it looked like samples made of RCG had better fatigue performance. In addition, it was discovered that the angle of internal friction in glass asphalt was much greater than that seen in conventional asphalt. This was because RCG particles had an angular shape, which made it easier for the parts to fit together. Behbahani et al. [70] found that following their investigations on samples that contained 10% recycled and crushed glass and 4% anti-stripping agents, they showed a much better resistance to humidity.

**Figure 11:**
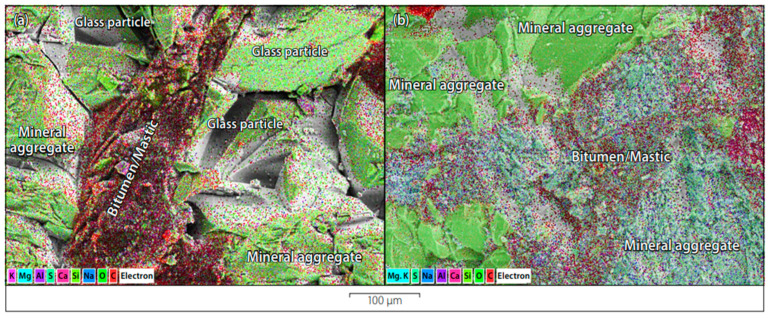
SEM of (**a**) glass-asphalt mix and (**b**) conventional mix [71].

T. B. George et al. [71] assessed the laboratory performance of a 10 mm average density asphalt wearing course that consisted of 15% recycled crushed glass in the following ways: (1) the effect of 1% hydrated lime; (2) the effect of 0.5% liquid anti-skid additive; and (3) the effect without the addition of an anti-skid additive. They evaluated the effectiveness of selected anti-skid additives to resist moisture damage. The TSR that was received from the modified Lottman test was used to assess the influence of these factors on the moisture susceptibility of the glass asphalt mix. This determination was confirmed by microscopic imaging analysis that was done on the modified Lottman samples that were tested. After this, the stiffness and permanent deformation qualities of the glass asphalt mix that displayed the best possible resistance to moisture damage were contrasted with those of a traditional asphalt mix that did not include broken glass. To define the stiffness of the material, the Huet-Sayegh model and a polynomial model were used, Figure 11.

Jony et al. [72] investigated in 2011 the use of 0.075 mm RCG powder in a proportion of 95% in hot-mix asphalt concrete as a substitute for ordinary Portland cement and limestone powder. This was done in lieu of the more conventional use of limestone powder and OPC. The experiment consisted of nine different combinations, each with three different types of filler (limestone powder, Portland cement, and RCG) and three different amounts of filler (4%, 7%, and 10%). Another study by Roaa H. Latief [73] indicates that the performance of glasphalt containing up to 20% RCG glass with a maximum particle size of 4.75 mm is superior compared to conventional asphalt mixtures.

### 3.5. Use of RCG in Geotechnical Applications

The use of waste glass in traditional geotechnical applications is often under-researched and limited to a few applications; this may be because of a lack of knowledge of its geotechnical and environmental properties. That’s because it’s impossible to reuse broken glass [74]. Numerous studies have shown RCG’s potential for use as a geomaterial since it has many of the same qualities as a natural aggregate [75]. Thoroughly cleaned glass can fully replace the role of natural granular components [76]. According to the American Society for Testing and Materials (ASTM), most cullet might be on par with clean sand.

Both structural and nonstructural fill applications benefit from the use of fine RCG. While RCG has several potential geotechnical applications, the most typical ones are drainage blankets, filter media, soil stabilizers, and backfill material for road pavements and embankments. In addition to its usage as a soil stabilizer, drainage blanket, and filtering medium are further geotechnical uses of RCG. Only a few studies have looked at RCG’s potential in geotechnical settings.

There have been many studies on the effects of fine RCG on various soils in recent years. Olufowobi et al. [77] conducted tests on clayey soil, varying the amounts of powdered RCG while keeping the cement content at 15%. They measured the effects on shear strength, CBR, and compaction. After being exposed to 5% RCG, it was found that the MDD of modified clay increased somewhat, from 25.37 to 25.90 kN/m^3^. At a concentration of 10% RCG, however, the shear strength metrics of modified clay improved most noticeably, with the friction angle also increasing from 15 to 17 degrees. Based on their results, the researchers concluded that the sweet spot for RCG is anywhere between five and ten percent, depending on the nature of the improvement to be made. Amiri et al. [78] investigated the influence of RCG on the geotechnical characteristics of kaolinite at doses ranging from 10% to 50% and particle sizes from 2.36 mm to 1.18 mm. The study’s results showed that up to a percentage of 50% replacement, RCG improved the friction angle, compressive strength, and MDD of kaolinite. When compared to other possible materials, RCG is a viable option for enhancing the geotechnical properties of cohesive soils. This alternative would be cheap, easy to get, and environmentally sound. The behavior of compaction and strength was investigated by Arabani et al. [79] using poorly graded sand stabilized with cement at 5% and 7% concentrations and RCG at 20%, 40%, and 60% concentrations. The RCG utilized had a particle size less than 12.7 mm in diameter. The research found that the CBR, compaction, and shear strength characteristics of modified sand significantly improved with the addition of 60% and 7% of RCG and cement, respectively. This was especially noticeable after the introduction of these two additives. In particular, the mixture of 60% RCG and 7% cement had a UCS that was 3.5 times higher than before. Canakci et al. [80] investigated the effects of RCG fine enough to pass through a sieve with a 75-m aperture on the clay’s density, toughness, and uniformity. There were 3%, 6%, 9%, and 12% RCG additions to the formula. Incorporating RCG into clay led to a sustained improvement in the material’s compressive strength. The pozzolanic activity of RCG at finer grain sizes may account for the observed increase in CBR. In addition, this study tested the effects of 3, 7, and 28 days of curing on the unconfined compressive strength (UCS) of RCG-clay samples. Results showed a significant correlation between the UCS of samples and curing time; however, the addition of 6% RCG yielded the greatest results in increasing the clay’s UCS.

**Figure 12:**
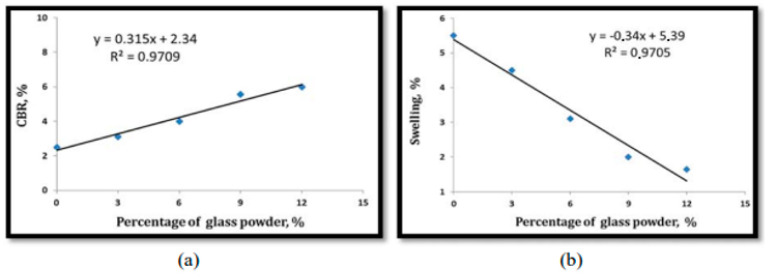
(**a**) Relationship between CBR and percentage of WSLGP, (**b**) Relationship between swelling and percentage of WSLGP [80].

**Figure 13:**
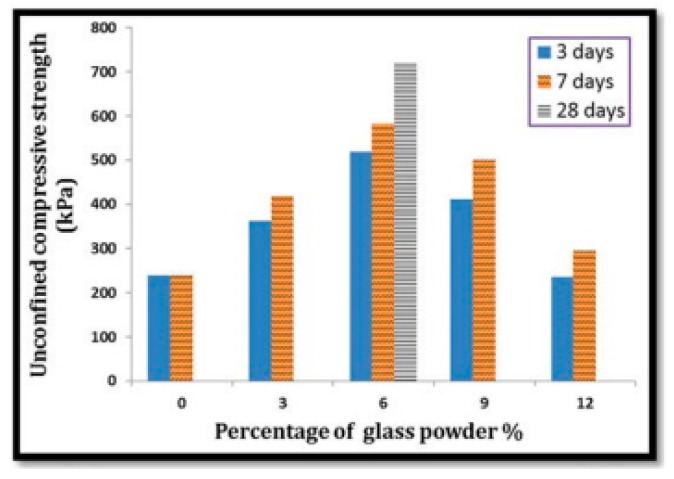
Effect of curing time on unconfined compressive strength of the samples [82].

Furthermore, the same study discovered that the addition of RCG increased MDD while concurrently decreasing the OMC of clay, Figure 12 and Figure 13 Khan et al. [81] investigated many geotechnical soil properties when RCG was combined at 4%, 8%, and 12%. For this project, we needed RCG with a particle size of less than 0.074 mm and a soil classification of CL-ML. In general, the findings demonstrated that the implementation of RCG was successful. Adding 4% RCG, for instance, significantly raised the CBR of soil by 11.26%, which was much greater than the other RCG addition rates. The comparison here is that incorporating RCG increased the soil’s resistance to erosion by increasing the soil’s friction angle. The results showed that 8% RCG produced the greatest improvement in soil friction angle. The increase is 1.12 times the amount before this component was included. 

Difani et al. [74] performed a study on the effect of RCG on the geotechnical behavior of biosolids and found the following: (Bio). The tests made use of both the pure and mixed versions of the ingredients. The preparation of the biosolids resulted in mixtures because of the varying RCG applications at various concentrations. The study found that mixes of RCG 40/Bio60, RCG 50/Bio50, and RCG 60/Bio40 improved geotechnical performance, particularly in terms of shear strength. Overall, the study’s findings suggested that mixtures of RCG and biosolids might effectively substitute natural aggregates in many geotechnical contexts, including as a material for filling embankments. The researchers arrived at this conclusion after discovering that mixtures of RCG and biosolids showed great promise for replacing natural aggregates in several geotechnical applications. Attom [82] investigated the potential of RCG to mitigate the swelling pressure from clayey soils. We employed high-plasticity clay for the base and remolded samples using two cement percentages (5 and 10 percent) and four RCG concentrations (2%, 5%, 7% and 10%). We used three different RCG particle sizes and a relative compaction level of 95% to form a matrix of soil and RCG. The swelling pressure of composites was studied by conducting trials with no swelling. Based on their results, the researchers concluded that mixing suitable amounts of RCG with cement would mitigate clay’s swelling potential.

Makowski and Rusenko [83] investigated the viability of using RCG (mean particle size 0.40 mm) as an alternative beach infill material. The study aimed to find viable alternatives to sand for use in reinforcing erodible coastlines. It was necessary to conduct chemical and biological tests on RCG to assess its viability as a replacement for sand in beach filling. The study suggests that RCG may be utilized instead of sand to both forestall the emergence of erosional hotspots and mitigate their effects [84].

To determine the effect of varying percentages of crushed glass on the geotechnical properties of soil mixtures, researchers have studied the physical properties of crushed glass for soil enhancement purposes, as in [85,86,87]. The glass was combined with processed dirt, often sand 25.4 mm or smaller. The results indicated that crushed glass had an average specific gravity of 2.5, a peak density of 16.2 kN/m^3^ with normal Proctor compaction, a maximum density of 18.1 kN/m^3^ with modified Proctor compaction, and an inner resistance of around 51 degrees. According to [85,86,87], who summarized the research of Dames and Moore, using crushed glass as a substitute for natural aggregates in construction is helpful.

### 3.6. The Use of RCG in the Manufacture of Tiles and Bricks

According to the published research, RCG has been employed in producing tiles and bricks in several experiments. RCG has been extensively examined as a fluxing material, notably as a substitute for feldspar, in the context of the tile industry. In porcelain stoneware tiles, Luz and Ribeiro [88] investigated the behavior of RCG as a potential replacement for traditional fluxing chemicals. The process of densification of the samples was sped up by RCG, which also resulted in an improvement in the samples’ decreased open porosity and absorption. However, certain unfavorable effects, such as greater shrinkage and higher closed porosity, were also seen at higher proportions of RCG. These effects were observed. According to the findings of the research as a whole, the dependability of stoneware tiles may be improved by adding a very tiny amount of fine RCG in addition to feldspar. According to the results published by other researchers who came to the same conclusions, RCG might be used as a partial alternative for feldspar in manufacturing porcelain sanitary ware. 

**Figure 14:**
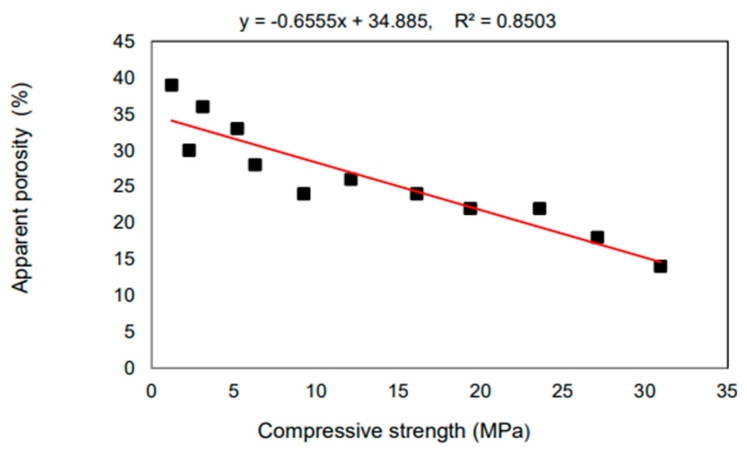
There is a correlation between the bricks’ compressive strength and their perceived porosity [83].

Aneke Frank Ikechukwu [89] reported the results of research on masonry bricks made from mixtures of Ordinary Portland Cement (OPC), recycled concrete (RCG), and fly ash (FA) in varying percentages. The masonry bricks were manufactured with 5%, 10%, and 15% additions of OPC to the raw materials, respectively, the total weight of FA and RCG combined. Compared to burnt clay bricks, which have an average compression strength of 3.8% greater, the manufactured bricks had a considerable resistance to compression. The compressive strength of all the bricks created for the research met the standards set by the South African National Standard Code SANS 227 (i.e., 7 MPa) for each load-bearing masonry brick. The scanning electron microscopy (SEM) study showed that the voids found in the microstructure of the 5% OPC bricks had low strength, the consequence of an incomplete pozzolanic reaction. This was determined by identifying the void spaces as the primary reason for the low strength. Additionally, the impacts of sulfate salt were considerably resisted on the surface of all the examined bricks, including FA and RCG. This was because the bricks included aluminosilicate compounds, which generated pozzolanic processes inside the matrix of the brick. Because of the increased strength formed in the bricks after they were produced, the tested bricks displayed brittle features in their stiffness. As a result of the percentages of RCG particles, this demonstrated the presence of a significant proportionality between the dynamic modulus and the ultrasonic pulse velocity (UPV). The coefficient of determination (R2) for this relationship was comparable to 90%. Figure 14.

The characteristics of RCG and white clay (thin film transistor-liquid crystal display) were investigated by Lin [90] as potential raw materials for the production of ceramic tiles Tile samples were made by incorporating 0–50% RCG into the ceramic mixture. Several tests, including bulk density, water absorption, fire shrinkage, and hardness, were administered to tile samples that had been changed. In general, the findings showed that using certain RCG as a raw material to manufacture ceramic tiles with adequate strength and microstructure qualities is feasible. This was proved by the findings. In a study that was quite similar, Kim et al. [91] (2016) looked into the possibility of using RCG, which stands for liquid crystal display, in ceramic tiles instead of feldspar. During the preparation of the samples, 40% of the feldspar in the ceramic mixture was substituted with RCG. In general, the findings of the experiments demonstrated that the tile samples that included RCG had a dense microscopic structure and better properties, such as a higher coefficient of thermal expansion and a lower water absorption rate. As a result, using RCG as a partial replacement for more traditional raw materials and fluxes in stoneware tile products is technically viable.

Several studies have evaluated the possibility of using RCG as an additive in the production of bricks. In research carried out by Loryuenyong et al. [92], the impacts of RCG on the mechanical and physical properties of clay bricks were investigated. The qualities of the clay bricks that resulted from the experiment were analyzed, and various percentages of RCG were included in the mixture. These percentages included 5%, 15%, 30%, and 45%. According to the conclusions of this study, if clay is mixed with anywhere from 15 to 30 percent RCG, it is possible to produce versatile bricks that are versatile enough to be used in various environments. However, the use of RCG at levels greater than 30% has the potential to negatively affect the performance of bricks in several ways. These methods include a drop in compressive strength and a modulus of rupture lower than expected. The findings of the research indicated that RCG concentrations of up to 30% may be applied to clayey bricks without risk.

Lin [93] made an effort to investigate the viability of ecobricks by substituting RCG for clay in their production (optical). The samples were prepared by firing them at temperatures ranging from 800 to 1000 degrees Celsius using varying percentages of RCG as a replacement: 0%, 10%, 20%, 30%, and 40%. This research showed that adding RCG to clay bricks may improve their characteristics, such as their specific gravity, water adsorption rate, and compressive strength. The same research, however, found that the presence of RCG proportionally increased the shrinkage in the bricks, especially at replacement proportions greater than 30%. This was particularly true when the replacement percentage was greater than 30%. It was determined that the best amount of RCG substituted for clay in bricks is around 30%.

Federico [94] conducted research to investigate how RCG affects the characteristics of burnt clay bricks. Eleven sets were constructed with A containing no glass, B containing 5% glass, C containing 5% glass, D containing 10% glass, E containing 10% glass, F containing 15% glass, G containing 15% glass, L containing no glass, X containing no glass, FL containing no glass, and FX containing no glass. Every set had a total of 30 different samples. Set A was designated as the test group, while the other groups were considered the control groups. A variable proportion of RCG was present in B, D, and F, but all of them had the same size of glass mesh. Each sample of C, E, and G had a unique proportion of RCG content while having the same glass mesh size. As a result, research was done to determine how the qualities of the bricks were affected by factors like the amount of RCG present and the size of the particles. In addition, samples L, X, FL, and FX were made to evaluate how the bricks were affected by the technique and the temperatures at which they were fired. To make the clay more malleable, lignosulfonate, in the amount of 20 milliliters, was mixed in with the water before the mixing process began. The results of the tests revealed that the percentage of RCG had a meaningful impact on the compressive strength of the material. It was determined that G (15.1%) had a compressive strength of 133.4 MPa, which was the highest value

When Dondi et al. [95] added 15% RCG to burned clay bricks, the bulk density rose by 1.1%. Another study came to a similar conclusion, reporting an increase of 2% when 25% RCG was added. Phonphuak et al. [96]. also showed that the brick specimens’ density rose by 3.5% when the bricks included 10% RCG. Additionally, research indicated that when less than 10% RCG (soda lime glass) was added to the bricks, the bulk density of the samples was minimally impacted by the quantity of waste glass in the mixture or the specified firing temperature [97]. This was the case even though the amount of RCG supplied to the bricks was less than 10%.

Additionally, it was noted that the bulk density increased dramatically with a rise in firing temperature when there was an addition of RCG content that was greater than 20%. The proportion can improve the quality of the bricks produced while allowing for acceptable shrinkage. Additionally, additional studies have demonstrated that RCG might produce bricks with the same or improved quality while reducing the required energy.

### 3.7. The Use of RCG in Water Filtration

To make polluted water suitable for consumption or use in some other capacity, it must first go through a process known as water filtration, which consists of removing particulates and other contaminants from the water using a filter medium. The selection of RCG as a filter medium is contingent on various criteria, such as the specific gravity of the particles as well as their form, size, and porosity [98].

In the treatment of septic tank effluent in intermittent recirculating biofilters, Hu and Gagnon [99] and in polishing filters for household wastewater Gill et al. [100]; Healy et al. 2010; Horan and Lowe [101], RCG has been evaluated as a wastewater filter medium. It has been claimed that RCG may retain 79% to 98% of total suspended particles in recirculating biofilters while also removing approximately 94% and 96% of BOD and ammonium nitrogen, respectively Elliott. The removal of 73% of the chemical oxygen requirement and 28% of the total nitrogen was reported for RCG by Gill et al. [102] when it was used as a tertiary filter medium. Researchers Salzmann and colleagues investigated using RCG as a tertiary filter medium for water treatment in municipal lagoons. They found that RCG had the same capacity for removing suspended particles as sand did and identical performance for ammonia and chemical oxygen demand parameters.

The outcomes of the study indicated that the use of RCG as a filter medium led to a decrease of 10% in the number of media required for the filtration process. The findings of the research indicated that RCG provided performance that was comparable to that of sand and, in some instances, even better. Similarly, the performance of RCG was evaluated alongside conventional sand media for high-rate filtration by the Clean Washington Center [103]. The usage of RCG was shown to enhance water clarity when compared to typical sand, as indicated by a 25% drop in the National Turbidity Unit (NTU) in conjunction with a 23% improvement in backwash efficiency. These results were seen in the research. The outcomes of this study indicate that the amount of RCG needed for filtering is 20% smaller than that of natural sand. This provides a greater economic benefit throughout the processing, shipping, and disposal operations since RCG requires less material overall. The effectiveness of RCG as a filter medium in treating municipal drinkable water was investigated by Evans et al. [104]. The quality of the water obtained from RCG was discovered to be equivalent to that of sand in terms of the number of particles, the turbidity, and the quantity of metals present. Therefore, RCG presents an opportunity to replace sand in water filtering applications while simultaneously improving the quality of the effluent produced.

Omer et al. [105] experimented with the replacement of coal with RCG. The use of broken recycled glass as a potential alternative to silica sand in dual-media filters has been researched to discover a suitable substitute. Experiments with inline filtration were carried out on a pilot scale using raw water obtained from three different water sources. The turbidity of the raw waters used in the experiments ranged from 6.0 to 14.0 NTU in each case. During the studies, two filter columns that were physically similar were run in parallel. The first filter was made up of 62.5 cm of silica sand and 41.5 cm of anthracite coal, while the second filter was made up of 62.5 cm of crushed recycled glass and 41.5 cm of anthracite coal. The combined bed depth of the two filters was 104 cm. The following is a list of the characteristics of the medium: The effective dimension of the glass is 0.77 mm, and its homogeneity coefficient is 1.41. The effective size of the sand is 0.79 mm, and the uniformity coefficient is 1.33. The effective size of coal is 1.45 mm, and the uniformity coefficient is 1.39.

When no coagulant was used; when 5 and 10 mg/L of alum were used; when 5 and 10 mg/L of ferric chloride were used; and when no coagulant was used. These are the five different times that the tests were carried out. The amount of filtering performed was at a rate of 11.5 m per hour (Table 5). We examined the turbidity, particle counts, and head losses as functions of time and then compared the findings. It was discovered that the following things are correct: Since the effluent turbidities and particle counts of the two filters were relatively comparable to one another, this indicates that the effluent quality was essentially the same when broken glass was used instead of silica sand as the media in the filter. In most of the tests, the filter constructed with broken glass performed superiorly to the filter that contained sand regarding the clean-bed head loss and overall head loss. Additionally, the filter created with broken glass had fewer severe head losses owing to clogging than the filter that included sand. It has been established that shattered glass may be an efficient alternative for silica sand in the process of dual-media filtration, which uses two different types of media [105].

Selda Yiğit Hunce et al. [106] conducted tests with three different media, namely silica sand, crushed recycled glass, and re-crushed recycled glass. The same sieved fractions, i.e., 0.85–0.71 mm, 1.00–0.85 mm, and 1.18–1.00 mm, were used for all materials. Performing tests with the same sizes of different materials allowed investigation of the effect of particle shape on filterability. The sphericity of the glass fractions was significantly lower than that of the sand medium, while lower differences in sphericity were observed between the crushed and RCG fractions. The tests showed that the glass fractions produced better filter performance than sand and silica.

### 3.8. Thermal Properties for Dwellings

The findings of a large number of research point to an upward trend in the biosphere’s mean temperature as a direct result of climate change. Because of this research, there has been a change in focus toward developing better waterproofing materials for glazing, particularly roofs. You might also use recycled domestic glass sand as an alternative form of insulation. In addition to its use in residential construction, particularly as a roofing material, it has been the subject of research, facilitating the extraction of alternative materials from the natural world.

**Figure 15:**
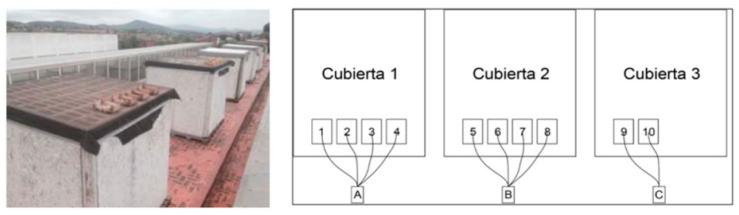
Presentation of the selected modules with the different broken glass boxes, positioned 1, 2 and 3 [107].

Researchers from the Autonomous University of Mexico, Alejandro Mata and Carlos Galvez [107], researched the most frequently utilized colors. Their findings were as follows: 60% of it is green, 25% of it is glass that is used for pharmaceuticals and soft drinks, 10% of it is exceptionally clear and is used most often for home usage (jars, glasses, cans, etc.), and 5% of it is amber or topaz that is used in pharmaceutical bottles of beer bottles. On a scale with finite increments, the Solar Reflectance Index (SRI) has the following values: the white layer has an emissivity of 0.9 and a reflectance of 0.8, while the black layer has an emissivity of 0.9 and a reflectance of 0.05. White, for instance, will heat up by 14.6 degrees Fahrenheit (8.1 degrees Celsius), whereas black will heat up by 90 degrees Fahrenheit (50 degrees Celsius). Therefore, the SRI will be computed using a scale of values that ranges from zero to one hundred. It measures the capacity to absorb heat and radiation, with a value of 100 indicating that the material absorbs the least amount of heat and radiation possible Figure 15 [107].

To achieve this goal, research was conducted on the roof of a school in Barcelona throughout June, July, and August. For the experiment, numerous wooden boxes filled with recycled glass sand of varying hues were positioned on the roof: 1. assorted crushed glass; 2. crushed topaz glass; 3. crushed household glass; 4. transparent flat crushed glass; 5. crushed decorative black glass; 6. decorative purple crushed glass tiles, 14 × 28 × 1.3 cm, 7. decorative crushed glass; 8. decorative crushed green gravel; 9. unglazed baked clay; 10. white limestone Table 6.

In comparison to traditional materials like limestone, gravel, or burned clay, the results of the experiment did not demonstrate a substantial decrease in temperature. The development of many little air chambers, which aid in the roof’s insulation, is one of the benefits of this design. The use of recycled glass sand is advantageous for the building sector in terms of cost savings since it is simple to acquire via the process of recycling, does not incur any expenses associated with extraction, is straightforward to process, and does not disrupt the insulating layer [107].

**Figure 16:**
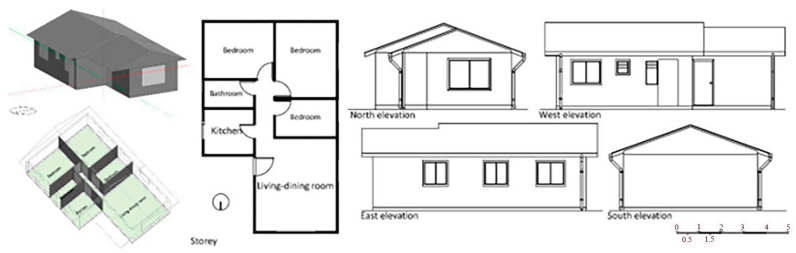
Presentation of the selected modules with the different broken glass boxes, positioned 1, 2 and 3 [108].

In the year 2020, Vicente Flores-Alés and Alexis Pérez-Fargallo [108] will employ mortar blocks that comprise either 25% (m25) or 50% (m50) thermally tested recycled glass aggregate. This suggests a perspective that is peripheral to the conventional approach, which is centered on the behavior of mechanical systems. The study method focuses on social housing Figure 16 and employs 21 models spread throughout Chile’s seven main climate zones. Thermal analysis is handled holistically using a technique based on analyzing periodic thermal transmittance, adaptive comfort levels, and energy consumption. This makes it easy to generalize this methodology to various building options.

When the findings of the thermal transmission are considered, it is possible to point out that for m50 solutions, it is decreased by 22%, the decay factor is decreased by 10%, and the time lag is increased by about one hour. In the meantime, places with a larger thermal amplitude between day and night, like 6 (Lonquimay) and 3 (Santiago), both in the winter and summer, demonstrate considerable reductions compared to standard solutions that do not include recycled glass aggregate.

Concerning the level of thermal comfort, we can conclude that an increase in the number of comfort hours is observed in all zones with the exception of zone 7 (Punta Arenas), with the m50 solution causing a rise of 199 h in zone 1 (Antofagasta). Zone 1 (Antofagasta), Zone 2 (Valparaiso), Zone 3 (Santiago), and Zone 6 (Lonquimay) are the ones that feel the effects of increased comfort hours and decreased distance from neutral temperature the most. Compared to the m25 solution, the m50 solution almost doubles the number of comfortable hours while cutting half the distance to the neutral temperature [108].

The most important discovery in terms of energy consumption is the decrease in peak demand in all thermal zones. This reduction ranges from a low of 0.1% (heating) with m25 in zone 7 to a high of 12.2% (cooling) with m50 in zone 1. The annual energy consumption in zones 1 (Antofagasta), 2 (Valparaiso), and 3 (Santiago) has decreased by up to 15%. Compared to m25, m50 results in a demand reduction of about fifty percent lower [108].

When it comes to the implementation of a building solution in a nation that makes use of recycled materials, this study is a starting point that should be respected in the decision-making process. The findings of the simulation need to be tested with genuine prototypes in the work that will be done in the future. To produce an implementation methodology for solutions for new construction that consider energy behavior, more study is required. This must be done keeping in mind the energy poverty levels of social housing and the fact that this cannot be accomplished in any other manner than with solutions that have a low environmental effect.

## 4. Potential Future Studies

Studies on heat-treated crushed and recycled glass focus on evaluating the thermal impact on the properties of recycled glass and the possibility of its use in various applications. This heat treatment involves heating recycled and crushed glass at high temperatures to improve its structure and property.

Some areas of research and development include: 

Thermal stability: The studies aim to evaluate the thermal behavior of recycled and crushed glass under the influence of high temperatures. Size, crystallization, fusion, or fragility changes are analyzed depending on the heat treatment applied.

Mechanical properties: How heat treatment affects the mechanical properties of recycled and crushed glass is investigated. Tensile strength, compressive strength, and impact strength are measured to determine whether heat treatment improves or impairs these properties.

Compatibility with other materials: The compatibility of recycled and crushed heat-treated glass with other materials used in various applications is examined. Adhesion, corrosion resistance, and joining behavior with metals or other materials are investigated to evaluate the possibilities of their use in composite products.

Specific Applications: Potential applications of heat-treated recycled and crushed glass are explored. These may include use in the construction industry, the production of packaging materials, glass fibers, or insulating materials.

Studies on heat-treated crushed and recycled glass are aimed at improving the efficiency of the glass recycling process and expanding its use in various fields. By understanding the properties and behavior of heat-treated recycled glass, sustainable and environmentally friendly solutions for glass waste management can be developed.

## Figures and Tables

**Table 1:** Percentage of recycled glass in different countries [7,8,9,10].

Nr.	Country	RCG%
01.	Belgium	96%
02.	Switzerland	94%
03.	Luxembourg	93%
04.	Netherlands	91%
05.	Netherlands	91%
06.	Norway	89%
07.	Germany	82%
08.	Italy	74%
09.	France	67%
10.	United Kingdom	61%
11.	Spain	57%
12.	USA	33%

**Table 2:** Analysis for glass powder [19].

Compound/Element	SiO_2_	Al_2_O_3_	Fe_2_O_3_	CaO	Na_2_O	MgO	K_2_O	SO_3_
Content (% weight)	72.58	1.47	0.85	10.49	12.54	0.61	0.4	0.2

**Table 3:** Chemical composition of soda-lime glass [23,24,25].

Type	SiO_2_	Al_2_O_3_	CaO	MgO	Fe_2_O_3_	Na_2_O	K_2_O	TiO_2_	SO_3_
Window	71.71	1.26	8.44	4.16	0.09	13.61	0.4	0.07	0.25
Containers	66–75	0.7–7	6–12	0.1–5	-	12–16	-	-	-
Light bulbs	73	1	5	4	-	17	-	-	-
Sheet	71–73	0.3	8–10	1–3.5	-	12–15	-	-	-
Float	73–74	-	8.8	3.7	-	13–15	0.2	-	-

**Table 4:** Physical properties of crushed waste glass [26,27,28].

General Properties	
Bulk density	1360 kg/m^3^
Specific gravity	2.4–2.8
Fineness modulus	0.43–3.29
Fineness modulus	84.4–94.7
Shape index (%)	30.5
Mechanical properties	
California bearing ratio (%)	50–75
Los Angeles value (%)	25–28
Friction angle	Crit = 38 (loose state)
Friction angle	Crit = 50–61 (dense state)
Thermal properties	
Thermal conductivity	0.7–1.3 W/m·K
Thermal heat capacity	850–950 J/kg·K
Thermal expansion coefficient	9.1–9.5 µstrain/°C

**Table 5:** The experimental matrix that was used for the sake of this study [104].

Omerli Water	Kağıthane Water	Hacı Osman Water
No coagulant	No coagulant	No coagulant
5 mg/L alum	5 mg/L alum	5 mg/L alum
10 mg/L alum	10 mg/L alum	10 mg/L alum
5 mg/L ferric	5 mg/L ferric	5 mg/L ferric
chloride	chloride	-
10 mg/L ferric	10 mg/L ferric	
chloride	chloride	

**Table 6:** Temperature measurement channels were used for the experiment [107].

Channel	RCG	°C
01.	R1—various crushed glass	25.09 °C
02.	R2—topaz crushed glass	17.38 °C
03.	R3—crushed glass	25.10 °C
04.	R4—transparent crushed flat glass	26.45 °C

## Data Availability

Not applicable.

## References

[1] Balta P. (1969). Glass and Its Current Applications.

[2] Gastev I.A., Rodin S.V. (1949). Glass Technology.

[3] Roychand R., Li J., De Silva S., Saberian M., Law D., Kumar P.B. (2021). Development of zero cement composite for the protection of concrete sewage pipes from corrosion and fatbergs. Resour. Conserv. Recycl..

[4] Saberian M., Li J., Perera S.T.A.M., Zhou A., Roychand R., Ren G. (2021). Large-scale direct shear testing of waste crushed rock reinforced with waste rubber as pavement base/subbase materials. Transp. Geotech..

[5] Global Alliance for Buildings and Construction, International Energy Agency, The United Nations Environment Programme (2019). 2019 Global Status Report for Buildings and Construction: Towards a Zero-Emission, Efficient and Resilient Buildings and Construction Sector.

[6] Saberian M., Perera S.T.A.M., Li J., Zhu J., Wang G. (2022). Effect of crushed glass on the shear behavior of recycled unbound granular aggregates incorporating crumb rubber. Int. J. Pavement Res. Technol..

[7] Harder J. (2018). Glass Recycling—Current Market Trends. Recovery. Viewed 16/10/2021. https://www.recovery-worldwide.com/en/artikel/glass-recycling-current-market-trends_3248774.html.

[8] Pickin J., Wardle C., O’Farrell K., Nyunt P., Donovan S. Australian National Waste Report 2018. Viewed 12/04/2021. https://www.environment.gov.au/system/files/resources/7381c1de-31d0-429b-912c-91a6dbc83af7/files/national-waste-report-2018.pdf.

[9] Ferdous W., Manalo A., Siddique R., Mendis P., Zhuge Y., Wong H.S., Lokuge W., Aravinthan T., Schubel P. (2021). Recycling of landfll wastes (tyres, plastics and glass) in construction—A review on global waste generation, performance, application and future opportunities. Resour. Conserv. Recycl..

[10] Deschamps J., Simon B., Tagnit-Hamou A., Amor B. (2018). Is open-loop recycling the lowest preference in a circular economy? Answering through LCA of glass powder in concrete. J. Clean. Prod..

[11] https://ecoteca.ro/evolutia-ambajelor-puse-pe-piata-si-valorificate-in-perioada-2009-2022-in-romania.html.

[12] Dhir R.K., Brito J.D., Ghataora G.S., Lye C.-Q. (2018). Chapter 8: Use of Glass Cullet in Road Applications. Sustainable Construction Materials.

[13] Poulikakos L.D., Papadaskalopoulou C., Hofko B., Gschösser F., Falchetto A.C., Bueno M., Arraigada M., Sousa J., Ruiz R., Petit C. (2017). Harvesting the unexplored potential of European waste materials for road construction. Resour. Conserv. Recycl..

[14] Plati C. (2019). Sustainability factors in pavement materials, design, and preservation strategies: A literature review. Constr. Build. Mater..

[15] Ali M., Arulrajah A., Disfani M., Piratheepan J. (2011). Suitability of using recycled glass—Crushed rock blends for pavement subbase applications. Geo-Front. Am. Soc. Civ. Eng..

[16] Arulrajah A., Ali M.M.Y., Disfani M.M., Piratheepan J., Bo M.W. (2013). Geotechnical Performance of Recycled Glass-Waste Rock Blends in Footpath Bases. J. Mater. Civ. Eng..

[17] Ali M.M.Y., Newman G., Arulrajah A., Disfani M. (2011). Application of recycled glass—Crushed rock blends in road pavements. Aust. Geomech. J..

[18] Bilondi P.M., Toufigh M.M., Toufigh V. (2018). Experimental investigation of using a recycled glass powder-based geopolymer to improve the mechanical behavior of clay soils. Constr. Build. Mater..

[19] Xiao R., Polaczyk P., Zhang M., Jiang X., Zhang Y., Huang B., Hu W. (2020). Evaluation of Glass Powder-Based Geopolymer Stabilized Road Bases Containing Recycled Waste Glass Aggregate. Transp. Res. Rec..

[20] Dinh H.L., Doh J.H., Liu J., Lu L., Song H., Park D. (2023). Comprehensive assessment of geopolymer concrete mechanical and environmental performance with glass cullet fine aggregates. J. Build. Eng..

[21] Edwards D., Schelling J. (1999). Municipal waste life cycle assessment: Part 2: Transport analysis and glass case study. Process Saf. Environ. Prot..

[22] Glass Packaging Institute (2010). Complete Life Cycle Assessment of North American Container Glass. http://www.gpi.org/sites/default/files/NAmerican_Glass_Container_LCA.pdf.

[23] Mohajerani A., Vajna J., Cheung T.H.H., Kurmus H., Arulrajah A., Horpibulsuk S. (2017). Practical recycling applications of crushed waste glass in construction materials: A review. Constr. Build. Mater..

[24] Sobolev K., Türker P., Soboleva S., Iscioglu G. (2007). Utilization of waste glass in ECO cement: Strength properties and microstructural observations. Waste Manag..

[25] Castro S., de Brito J. (2013). Evaluation of the durability of concrete made with crushed glass aggregates. J. Clean. Prod..

[26] Ooi P.S., Li M.M., Sagario M.L., Song Y. (2008). Shear strength characteristics of recycled glass. Transp. Res. Rec..

[27] Disfani M.M., Arulrajah A., Bo M.W., Hankour R.J.W.M. (2011). Recycled crushed glass in road work applications. Waste Manag..

[28] Abdallah S., Fan M. (2014). Characteristics of concrete with waste glass as fine aggregate replacement. Int. J. Eng. Tech. Res..

[29] Zamora-Castro S.A., Salgado-Estrada R., Sandoval-Herazo L.C., Melendez-Armenta R.A., Manzano-Huerta E., Yelmi-Carrillo E., Herrera-May A.L. (2021). Sustainable Development of Concrete through Aggregates and Innovative Materials: A Review. Appl. Sci..

[30] Adaway M., Wang Y. (2015). Recycled glass as a partial replacement for fine aggregate in structural concrete—Effects on compressive strength. Electron. J. Struct. Eng..

[31] Ali E.E., Al-Tersawy S.H. (2012). Recycled glass as a partial replacement for fine aggregate in self compacting concrete. Constr. Build. Mater..

[32] Borhan T.M. (2012). Properties of glass concrete reinforced with short basalt fiber. Mater. Des..

[33] Taha B., Nounu G. (2009). Utilizing waste recycled glass as sand/cement replacement in concrete. J. Mater. Civ. Eng..

[34] Rajeev D., Jonathan J., Christophe G., Ayodele O. (2021). Exploring the Effects of the Substitution of Freshly Mined Sands with Recycled Crushed Glass on the Properties of Concrete. Appl. Sci..

[35] Malik M.I., Manzoor A., Ahmad B., Asima S., Ali R., Bashir M. (2014). Positive potential of partial replacement of fine aggregates by waste glass (<600 Microm) in concrete. Int. J. Civ. Eng. Technol..

[36] Limbachiya M.C. (2009). Bulk engineering and durability properties of washed glass sand concrete. Constr. Build. Mater..

[37] Chen C.H., Wu J.K., Yang C.C. (2006). Waste E-glass particles used in cementitious mixtures. Cem. Concr. Res..

[38] Lee G., Poon C.S., Wong Y.L., Ling T.C. (2013). Effects of recycled fine glass aggregates on the properties of dry–mixed concrete blocks. Constr. Build. Mater..

[39] Oliveira R., de Brito J., Veiga R. (2015). Reduction of the cement content in rendering mortars with fine glass aggregates. J. Clean. Prod..

[40] Ismail Z.Z., Al-Hashmi E.A. (2009). Recycling of waste glass as a partial replacement for fine aggregate in concrete. Waste Manag..

[41] Rui X., Baoshan H., Hongyu Z., Yuetan M., Xi J. (2022). A state-of-the-art review of crushed urban waste glass used in OPC and AAMs (geopolymer): Progress and challenges. Clean. Mater..

[42] Polley C., Cramer S.M., de la Cruz R. (1998). Potential for using waste glass in Portland cement concrete. J. Mater. Civ. Eng..

[43] Turgut P., Yahlizade E.S. (2009). Research into concrete blocks with waste glass. Int. J. Environ. Sci. Eng..

[44] Batayneh M., Marie I., Asi I. (2007). Use of selected waste materials in concrete mixes. Waste Manag..

[45] Park S.B., Lee B.C., Kim J.H. (2004). Studies on mechanical properties of concrete containing waste glass aggregate. Cem. Concr. Res..

[46] Çelik A.İ., Özkılıç Y.O., Zeybek Ö., Karalar M., Qaidi S., Ahmad J., Burduhos-Nergis D.D., Bejinariu C. (2022). Mechanical Behavior of Crushed Waste Glass as Replacement of Aggregates. Materials.

[47] Shehata I., Varzavand S., Elsawy A., Fahmy M. The use of solid waste materials as fine aggregate substitutes in cementitious concrete composites. Proceedings of the 1996 Semisesquicentennial Transportation Conference Proceedings.

[48] Saribiyik M., Piskin A., Saribiyik A. (2013). The effects of waste glass powder usage on polymer concrete properties. Constr. Build. Mater..

[49] Topcu I.B., Canbaz M. (2004). Properties of concrete containing waste glass. Cem. Concr. Res..

[50] Arivalagan V.S.S. (2021). Sethuraman, Experimental study on the mechanical properties of concrete by partial replacement of glass powder as fine aggregate: An environmentally friendly approach. Mater. Today Proc..

[51] Provis J.L. (2018). Alkali-activated materials. Cem. Concr. Res..

[52] Xiao R., Jiang X.I., Zhang M., Polaczyk P., Huang B. (2020). Analytical investigation of phase assemblages of alkali-activated materials in CaO-SiO_2_-Al_2_O_3_ systems: The management of reaction products and designing of precursors. Mater. Des..

[53] Wang Y., Xiao R., Hu W., Jiang X.i., Zhang X., Huang B. (2021). Effect of granulated phosphorus slag on physical, mechanical and microstructural characteristics of Class F fly ash based geopolymer. Constr. Build. Mater..

[54] Zhou S., Lu C., Zhu X., Li F. (2020). Preparation and characterization of high-strength geopolymer based on BH-1 lunar soil simulant with low alkali content. Engineering.

[55] Xiao R., Ma Y., Jiang X., Zhang M., Zhang Y., Wang Y., Huang B., He Q. (2020). Strength, microstructure, efflorescence behavior and environmental impacts of waste glass geopolymers cured at ambient temperature. J. Clean. Prod..

[56] Vafaei M., Allahverdi A. (2017). High strength geopolymer binder based on waste-glass powder. Adv. Powder Technol..

[57] Si R., Dai Q., Guo S., Wang J. (2020). Mechanical property, nanopore structure and drying shrinkage of metakaolin-based geopolymer with waste glass powder. J. Clean. Prod..

[58] Si R., Guo S., Dai Q., Wang J. (2020). Atomic-structure, microstructure and mechanical properties of glass powder modified metakaolin-based geopolymer. Constr. Build. Mater..

[59] Cyr M., Idir R., Poinot T. (2012). Properties of inorganic polymer (geopolymer) mortars made of glass cullet. J. Mater. Sci..

[60] Bilondi M.P., Toufigh M.M., Toufigh V. (2018). Using calcium carbide residue as an alkaline activator for glass powder–clay geopolymer. Constr. Build. Mater..

[61] Arabani M. (2011). Effect of glass cullet on the improvement of the dynamic behaviour of asphalt concrete. Constr. Build. Mater..

[62] Turkey F.A., Beddu S.B., Ahmed A.N., Al-Hubboubi S.K. (2022). Effect of high temperatures on the properties of lightweight geopolymer concrete based fly ash and glass powder mixtures. Case Stud. Constr. Mater..

[63] Derinpinar A.N., Karakoç M.B., Özcan A. (2022). Performance of glass powder substituted slag based geopolymer concretes under high temperature. Constr. Build. Mater..

[64] Jiang X., Zhang Y., Zhang Y., Ma J., Xiao R., Guo F., Bai Y., Huang B. (2023). Influence of size effect on the properties of slag and waste glass-based geopolymer paste. J. Clean. Prod..

[65] Çelik A.İ., Tunç U., Bahrami A., Karalar M., Mydin M.A., Alomayri T., Özkılıç Y.O. (2023). Use of waste glass powder toward more sustainable geopolymer concrete. J. Mater. Res. Technol..

[66] Shafabakhsh G.H., Sajed Y. (2014). Investigation of dynamic behavior of hot mix asphalt containing waste materials; case study: Glass cullet. Case Stud. Construc. Mater..

[67] Arabani M., Mirabdolazimi S.M., Ferdowsi B. (2012). Modeling the fatigue behaviors of glasphalt mixtures. Sci. Iran..

[68] Airey G.D., Collop A.C., Thom N.H. Mechanical performance of asphalt mixtures incorporating slag and glass secondary aggregates. Proceedings of the 8th Conference on Asphalt Pavements for Southern Africa, CAPSA’04.

[69] Su N., Chen J.S. (2002). Engineering properties of asphalt concrete made with cycled glass. Resour. Conserv. Recycl..

[70] Behbahani H., Ziari H., Kamboozia N., Khaki A.L., Mirabdolazimi S.M. (2015). Evaluation of performance and moisture sensitivity of glasphalt mixtures modified with nanotechnology zycosoil as an anti-stripping additive. Constr. Build. Mater..

[71] George T.B., Anochie-Boateng J.K., Jenkins K.J. (2020). Laboratory performance and modelling behaviour of hot-mix asphalt with recycled crushed glass. J. South Afr. Inst. Civ. Eng..

[72] Jony H.H., Al-Rubaie M.F., Jahad I.Y. (2011). The effect of using glass powder filler on hot asphalt concrete mixtures properties. Eng. Technol. J..

[73] Latief R.H. (2019). Evaluation of the Performance of Glasphalt Concrete Mixtures for Binder Course. Int. J. Adv. Sci. Eng. Inf. Technol..

[74] Disfani M., Arulrajah A., Ali M., Bo M. (2011). Fine recycled glass: A sustainable alternative to natural aggregates. Int. J. Geotech. Eng..

[75] Landris T. (2007). Recycled Glass and Dredged Materials.

[76] Dhir R.K., de Brito J., Ghataora G.S., Lye C.Q. (2018). Sustainable Construction Materials: Glass Cullet.

[77] Olufowobi J., Ogundoju A., Michael B., Aderinlewo O. (2014). Clay soil stabilization using powdered glass. J. Eng. Sci. Technol..

[78] Amiri S.T., Nazir R., Dehghanbanadaki A. (2018). Experimental study of geotechnical characteristics of crushed glass mixed with kaolinite soil. Int. J. Geomate..

[79] Arabani M., Sharafi H., Habibi M.R., Haghshenas E. (2012). Laboratory evaluation of cement stabilized crushed glass-sand blends. Electron. J. Geotech. Eng..

[80] Canakci H., Aram A., Celik F. (2016). Stabilization of clay with waste soda lime glass powder. Procedia Eng..

[81] Khan M.S., Tufail M., Mateeullah M. (2018). Effects of waste glass powder on the geotechnical properties of loose subsoils. Civ. Eng. J..

[82] Attom M. The use of waste glass material to control soil swelling pressure. Proceedings of the International Conference on Technological Challenges for Better World.

[83] Makowski C., Rusenko K. (2007). Recycled glass cullet as an alternative beach fill material: Results of biological and chemical analyses. J Coast. Res..

[84] Kazmi D., Williams D.J., Serati M. (2020). Waste glass in civil engineering applications—A review. Int. J. Appl. Ceram. Technol..

[85] Moore D. (1993). Glass Feedstock Evaluation Project: Reports for Tasks 1 through 5.

[86] Moore D. (2014). Municipal Solid Waste Generation, Recycling, and Disposal in the United States Tables and Figures for 2012.

[87] Shin C.J., Sonntag V. (1994). Using recovered glass as construction aggregate feedstock. Transp. Res. Rec..

[88] Luz A., Ribeiro S. (2007). Use of glass waste as a raw material in porcelain stoneware tile mixtures. Ceram. Int..

[89] Ikechukwu A.F., Onyelowe K.C. (2021). Environmental sustainability of fly ash and recycled crushed glass blends: An alternative to natural clay for masonry bricks production. Int. J. Appl. Sci. Eng..

[90] Lin K.-L. (2007). Use of thin film transistor liquid crystal display (TFTLCD) waste glass in the production of ceramic tiles. J. Hazard Mater..

[91] Kim K., Kim K., Hwang J. (2016). Characterization of ceramic tiles containing LCD waste glass. Ceram. Int..

[92] Loryuenyong V., Panyachai T., Kaewsimork K., Siritai C. (2009). Effects of recycled glass substitution on the physical and mechanical properties of clay bricks. Waste Manag..

[93] Lin K.-L. (2007). The effect of heating temperature of thin film transistor-liquid crystal display (TFT-LCD) optical waste glass as a partial substitute partial for clay in eco-brick. J. Clean. Prod..

[94] Chidiac S.E., Federico L.M. (2007). Effects of Waste Glass Addition on the Properties of Fired Clay Brick. Can. J. Civ. Eng..

[95] Dondi M., Guarini G., Raimondo M., Zanelli C. (2009). Recycling PC and TV waste glass in clay bricks and roof tiles. Waste Manag..

[96] Phonphuak N., Kanyakam S., Chindaprasirt P. (2016). Utilisation of waste glass to enhance physical–mechanical properties of fired clay brick. J. Clean. Prod..

[97] Sarmeen Akhtar U., Moniruz Zaman M., Islam M.S., Nigar F., Hossain M.K. (2017). Effect of different types of glasses as fluxing agent on the sintering temperature of bricks. Trans. Indian Ceram. Soc..

[98] Dheyaa M.A.M.A.-T., Zwayen M.A.-M. (2016). Reusing pulverized solid wastes glass as a filtration medium. Iraqi J Mech Mater Eng..

[99] Hu Z.F., Gagnon G.A. (2006). Impact of filter media on the performance of full-scale recirculating biofilters for treating multi-residential wastewater. Water Res..

[100] Gill L.W., Veale P.L., Murray M. (2011). Recycled glass compared to sand as a media in polishing filters for on-site wastewater treatment. Water Pract. Technol..

[101] Horan N.J., Lowe M. (2007). Full-scale trials of recycled glass as tertiary filter medium for wastewater treatment. Water Res..

[102] Gill L., Doran C., Misstear D., Sheahan B. (2009). The use of recycled glass as a filter media for on-site wastewater treatment. Desalin. Water Treat..

[103] Clean Washington Center (1998). Evaluation of Recycled Crushed Glass sand Media for High-Rate Sand Filtration.

[104] Evans G., Dennis P., Cousins M., Campbell R. (2002). Use of recycled crushed glass as a filtration medium in municipal potable water treatment plants. Water Sci. Technol. Water Supply.

[105] Soyer E., Akgiray Ö., Eldem N.Ö., Saatçı A.M. (2012). On the Use of Crushed Recycled Glass Instead of Silica Sand in Dual-Media Filters. CLEAN Soli Air Water.

[106] Selda Y.H., Elif S., Ömer A. (2018). On the backwash expansion of graded filter media. Powder Technol..

[107] Christian-Jose L.S., Oriol M.R., Joan-Lluis Z.M. (2017). Evaluation of the temperature of the waterproofing membrane when a layer of recycled crushed glass finish is used on flat roofs to protect from sunlight. Cent. Innov. Technol. Policy Res. Energy Procedia.

[108] Vicente F.A., Alexis P.F., Jesús A.P.A., Carlos R.B. (2020). Effect on the Thermal Properties of Mortar Blocks by Using Recycled Glass and Its Application for Social Dwellings. Energies.

